# Infectious Disease: Dilemma for Trachoma Treatment?

**Published:** 2007-01

**Authors:** Julia R. Barrett

The WHO estimates that trachoma has blinded approximately 8 million people worldwide and threatens the vision of 84 million more, primarily in developing countries. Now a report in the 27 September 2006 issue of *JAMA* claims that treatment with azithromycin, a cornerstone in the WHO’s strategy to eliminate trachoma, may hinder development of protective immunity and eventually lead to increased disease prevalence. Some experts in trachoma control, however, disagree that the study data support such a conclusion.

Trachoma arises from repeated eye infection with *Chlamydia trachomatis*, spread by contact with infected persons and by flies. The WHO implemented its SAFE strategy—surgery (S), antibiotics (A), face washing (F), and environmental improvements (E)—in 1997. Community-wide dosing with treatments such as oral azithromycin significantly reduces infection rates. However, reemergence of infection after treatment is common.

The *JAMA* study, led by postdoctoral fellow Berna Atik of the Children’s Hospital Oakland Research Institute (CHORI) in California, compared three multi-village communes in Vietnam, each receiving a different SAFE-based treatment: surgery only, surgery and antibiotics, or all four components. Compared to the S commune, the SA and SAFE communes had significantly higher reinfection rates two years after final antibiotic treatment.

“Our finding that there was reemergence of infection isn’t surprising, but no one had performed a longitudinal analysis of risk factors for reemergence, including new infection, reinfection, or continuing infection,” says coauthor Deborah Dean, a senior scientist at CHORI.

According to Thomas Lietman, an associate professor of ophthalmology at the University of California, San Francisco, and coauthor of a review of the study to be published in the April 2007 *Archives of Ophthalmology*, the study reflects trachoma researchers’ concerns about immunity, but it’s inadequate to show a problem exists. “One of the things we really should be looking for in our trachoma programs is if there’s a loss of immunity, but they didn’t . . . account for just chance variation between the different communities,” he says.

This reaction puzzles Dean. “There was very little variation among the villages or communes as detailed in the article,” she says. “The two statistical models we used for longitudinal analyses are extremely robust and took into consideration variation in infection for all villages and communes at each six-month time point of the study.”

Paul Emerson, technical director of the Trachoma Control Program at the Carter Center in Atlanta, is concerned that antibiotic treatment may now mistakenly be considered counterproductive. “The concern I have is that [this study] will actually reduce the small amount of support going to trachoma control programs,” he says. Dean responds that the research team supports the SAFE program. However, she adds, “The ‘A’ component needs to be further evaluated longitudinally for its risks and benefits, especially with published evidence of chlamydial resistance to azithromycin.”

## Figures and Tables

**Figure:**
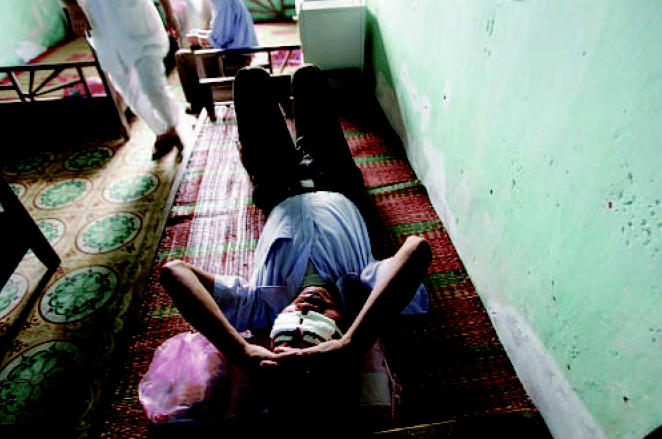
A Vietnamese man rests after eye surgery for trachoma.

